# On the Use of Ag/AgCl Reference Electrode in Silicon Nanowire Field Effect Transistors for pH and Biosensing Applications

**DOI:** 10.3390/s26030857

**Published:** 2026-01-28

**Authors:** Valérie Stambouli, Katell Aldrin, Edwige Bano

**Affiliations:** 1 Laboratoire des Matériaux et du Génie Physique, Université Grenoble Alpes, Centre National de la Recherche Scientifique, Institut Polytechnique de Grenoble, 38016 Grenoble, France; 2 Université Marie et Louis Pasteur, CNRS, Institut FEMTO-ST, F-25000 Besançon, France; katell.aldrin@femto-st.fr; 3 Centre de Radiofréquences, Optique et Micro-Nanoélectronique des Alpes, Université Grenoble Alpes, Centre National de la Recherche Scientifique, Institut Polytechnique de Grenoble, 38016 Grenoble, France; edwige.bano@grenoble-inp.fr

**Keywords:** reference electrode, Si NWFET, Ag/AgCl, pH, biosensors

## Abstract

Silicon nanowire field-effect transistors (Si NWFETs), thanks to their highly efficient integration into numerous electronic devices, represent promising tools for pH measurements and molecular biosensing due to their high sensitivity to ionic charges on their surface. To obtain a highly sensitive, stable, and low-noise signal in liquid media, the use of a reference electrode is necessary. Among potential reference electrodes, Ag/AgCl electrodes have proven to be the easiest to miniaturize and, consequently, the most widely used in Si NWFETs. However, their integration in such devices remains complex. The choice of the type of Ag/AgCl reference electrode and its positioning vary considerably depending on the device configuration and the analyte, and whether the measurements are carried out in dynamic or static environments. Here, we report a consolidated overview focused on the reference electrode implementation in Si NWFETs, presenting the advantages and disadvantages of different strategies encountered, and illustrating our points with several examples of miniaturized electrochemical biosensors and ion-sensitive field-effect transistors. Finally, in order to understand the effects of the Ag/AgCl electrode in Si NWFETs and its influence on the polarization of the device or the analyte, a comparative study of different device configurations from the literature is also presented.

## 1. Introduction

Electrochemical biosensors, like optical biosensors, constitute a widespread class of biosensors [1]. The main advantage lies in the ability to directly read the transduction signal on a laptop or mobile phone, while also allowing for the monitoring of its evolution over time. This is crucial for many routine applications, including ex vivo and in vivo monitoring of biomarkers, such as neurotransmitters [2]. The most widespread and well-known electrochemical biosensor is the glucose sensor [3], whose main application is in commercially available glucose patches for routine testing in diabetic patients. In this case, the patient can directly monitor their blood glucose levels on their mobile phone in preparation for insulin injections.

Electrochemical biosensors can be classified into two categories according to the mode of electrochemical transduction during biochemical recognition. The first category relies on charge transfer linked to a redox reaction, while the second relies on a change in surface conductivity induced by an electric field. Both categories require a set of three electrodes with distinct roles. The first category includes conventional electrochemical biosensors, for which measurements are performed in a three-electrode cell. This cell comprises (i) a conductive working electrode (WE) on which bioreceptors are immobilized, (ii) a counter electrode (CE), and (iii) a reference electrode (RE). The latter is often based on Ag/AgCl electrode. The key role of the RE is to provide a stable potential to which the other potentials can be referenced. Indeed, a redox reaction occurs at the surface of the working electrode (WE), inducing a charge transfer between an electroactive molecule (target analyte, immobilized enzyme, or redox mediator) and this electrode. This system is characterized by a known redox potential, the value of which is controlled by the reference electrode. Therefore, the use of a reference electrode is mandatory and systematic for these measurements. Significant research is currently underway to optimize both performance and portability. On the side, the sensitivity—conductivity and electrocatalytic activity properties—of the working electrode is enhanced using various conductive nanomaterials such as carbon nanotubes [4], graphene [5,6], gold nanoparticles [7], or dichalcogenures [8]. This enables the obtention of highly developed conductive surfaces with high densities of immobilized bioreceptor molecules [9]. On the other side, the design of the three-electrode cell trends toward miniaturization, where the Ag/AgCl reference electrode is obtained by printing techniques [10]. Today, portable and disposable electrochemical biosensors are available.

**Figure 1:**
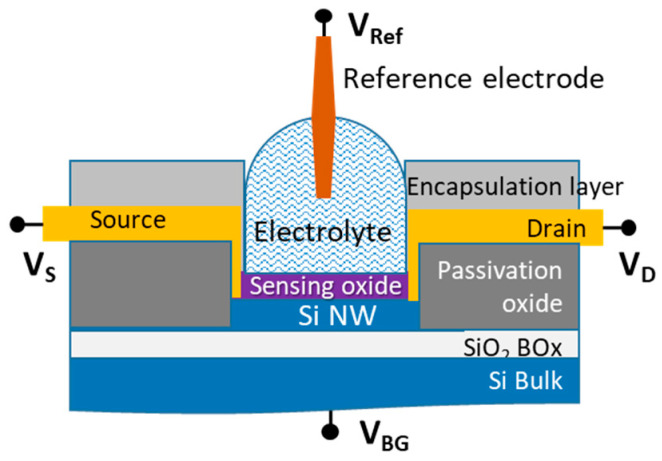
Generic cross-section schematic of a Si NW-based ISFET. The sensing surface is the Si nanowire that is covered by a thin top oxide (gate oxide) sensing film (for instance, SiO_2_, HfO_2_, Al_2_O_3_, etc.) in contact with the electrolyte. Generally, the Si NW originates from nanoline etching in a thin Si film of SOI wafer, including a buried oxide layer (BOx) on bulk Si.

The second category of electrochemical biosensors comprises semiconductor-based field effect devices (FEDs). In this category, unlike the systematic use of the Ag/AgCl reference electrode in conventional electrochemical sensors, like discussed above, its implementation is more complex and can sometimes be questionable, as will be detailed throughout this paper. The principle of the FED takes into account that most of biological molecules are charged. The electrostatic field effect affects the potential at the electrolyte/insulator/semiconductor interface and, in turn, modulates the charge carrier density in the semiconductor channel [11]. FED-based biosensors comprise several kinds of devices. The simplest in terms of layout—being both easy and cost effective—are capacitive electrolyte–insulator–semiconductor (EIS) structures [12]. The other typical devices are Si ion-sensitive field effect transistors (ISFETs) [13] and their nanoscale versions (Si NWFETs) [14], as illustrated in Figure 1. The sensing mechanism is enhanced, since the surface charges can efficiently gate the entire volume of the Si nanochannel.

Si NWFETs present many advantages. They benefit from high miniaturization, portability, and mass production at a low cost due to their fabrication process, which is compatible with the complementary metal oxide semiconductor (CMOS) process integrated circuit (IC) fabrication procedure. Regarding the setup of electrodes in a standard MOSFET (metal oxide semiconductor FET) device, three electrodes are involved with different configurations depending on the application. On the one side, for purely microelectronic applications, the voltage applied to the metal back-gate electrode [15] controls the current flow in the Si semiconducting channel between the source and drain metal electrodes. On the other side, in the MOSFET in contact with a liquid medium for pH measurements or biosensing applications—thus becoming an ISFET—there are typically three cases in the literature. One is to simply keep the back-gate electrode [16,17,18]. In a second case, the back-gate electrode is replaced by a Ag/AgCl reference electrode located in the liquid [15]. This gate reference electrode is often referred to as the top-gate, front-gate, or liquid-gate [11]. These devices are also sometimes called the electrolyte-gate FET (EGFET), not to be confused with the extended-gate FET, which is also referred to as the EGFET [19,20]. Finally, in a third case, both the back-gate electrode and the Ag/AgCl reference electrode are used. This last case is called a dual-gate configuration and will be described further in this paper. At this stage, it is clear that the use of a Ag/AgCl reference electrode is not systematic in Si NWFET devices. Additionally, in papers where a Ag/AgCl electrode is involved, various methods of using it are reported, making it difficult to describe the landscape of Ag/AgCl reference electrode usage in Si NWFETs. Before going further in the overview of the various configurations, it is useful to provide a short and brief history of Si NWFETs in biosensing, since there are some similarities with the evolution of three-electrode electrochemical cells in conventional electrochemical biosensors.

Historically, the first FED device was an ISFET used for pH sensing, which was introduced by P. Bergvelt in the 1970s [21,22]. As a MOSFET device with the 2D Si sensing layer directly in contact with the electrolyte, the protonation and deprotonation of the top SiO_2_ layer occurs as a function of the solution’s pH and according to the site binding model [23]. This results in a modulation of the Si channel conductance, since the binding of H^+^ ions induce a surface charge, which is balanced by a spatial rearrangement of the ions in the electrolyte. The resulting potential drop at the sensing interface is transduced into a shift in the threshold voltage (Vth) of the ISFET as a function of pH. The sensitivity, expressed in the mV/pH unit, depends on the nature of the oxide top-layer and the device characteristics, as will be reported further. Later, in 1997, such ISFETs were used for the first time for the FET detection of negatively charged DNA sequence hybridization [24] using impedance spectroscopy. In the two last decades, many works have aimed to enhance the performance/sensitivity of ISFETs. Thus, similarly to the improvements to the characteristics of the working electrodes in the case of conventional electrochemical sensors, as mentioned previously, enhancements to the electrical and morphological characteristics of the sensitive semiconducting Si material were performed. Notably, the nanostructuration of classical 2D Si channel films into arrays of Si nanowires with well-defined 1D shapes allowed for the development of the surface/volume ratio, thus enhancing the sensitivity of Si NWFET sensors [25,26]. Cui et al. were the first group to execute this geometry in 2001, using bottom-up vapor–liquid–solid (VLS)-grown Si NWs for pH sensing, as well as streptavidin protein and Ca^2+^ detection [27]. Relatively soon after, top-down fabricated Si NWFETs were introduced as sensing platforms in order to address the problems of placement, integration, and reproducibility encountered with bottom-up materials [28,29,30,31]. Since then, extensive works have been carried out on various kinds of Si NWFETs for biosensing. They aim at making the fabrication easier by enhancing the characteristics, performances, stabilities, and sensing signal reproducibility of Si NWFET devices while expanding the application fields. The progress of the work carried out in this domain is extensively reported in numerous review articles [11,32,33,34,35,36,37,38,39].

Like in the case of conventional electrochemical sensors, it is necessary to maintain a stable electrolyte potential for the reliable electrical detection of charged species at the Si NW surface. Indeed, if this potential changes, it will affect the potential distribution around the Si NW and the signal detected by the Si NWFET will no longer be due solely to a surface binding reaction [40,41]. Thus, for accurate and reproducible sensing measurements, a reference liquid electrode is required to provide a stable electrolyte potential. A good reference electrode for biosensing or pH sensing applications is one whose interfacial potential does not change significantly compared to the surface potential of the bioFET and which is stable over time [34,40,41]. With the emergence of Lab-on-Chip technologies, the miniaturization process of Si NWFET-based biosensing devices is required for increased accessibility for portability and on-site use. For instance, the recently reported Lab-on-Skin^TM^ multi-sensing system uses arrays of functionalized fully depleted Si on insulator (FD SOI) FET sensors with liquid-gates [42]. They are able to simultaneously detect pH, Na^+^, and K^+^ concentrations in sweat in real time [43,44]. Therefore, the miniaturization and integration of the reference electrode as part of the whole measurement system and its positioning close to the sensing area become major challenges in implementing such miniaturized biochemical measurements of Si NWFETs [15].

However, it is striking to note that the specific part related to the reference electrode is rarely discussed in detail, despite the increasing number of papers on the technology of Si NWFET-based biosensors—with huge efforts made toward integration strategies—and the impact of the fabrication processes and functionalization aspects on the sensor to sensor variations [38]. Indeed, finding resources on the fabrication, type, integration, size, positioning, and biasing of the different types of Ag/AgCl reference electrodes in biosensing NWFET devices has proven difficult. Only a few reviews reporting on this can be cited. For instance, in a review paper, L. Mu et al. [31] summarized the principles of CMOS-compatible Si NWFET nanobiosensors, such as the recent developments in device fabrication, the different biosensing applications, and the techniques of surface functionalization as well as fluid integration. They also referred to the major issues that need to be considered so to enable advancements in these types of devices. One of these issues is the simple and reliable integration of a reference electrode. The integration of a reference electrode is an essential yet non-trivial component of fluidic integration for Si NWFETs [31]. Another review by M. Shinwary et al. [45] presents the basic electrochemistry and thermodynamics of different kinds of reference electrodes. Moreover, their use in both electrochemical and ISFET biosensors are reported, emphasizing the issues related to electrode integration and miniaturization that can affect the long-term stability of the electrode as well as the performance of the biosensor. In a critical review published in 2017, B.M. Lowe et al. [11] reported on the broad diversity of reference electrode setups used in FET sensors in the literature. Notably, among the 37 publications identified by the authors, 19% used a Ag/AgCl pseudo-reference electrode, 11% used a conventional Ag/AgCl reference electrode, 27% utilized only a back-gate, and 21% did not publish their setup (or it was ambiguously presented). Additionally, in some papers, the results of electrical FET detection of biomolecules are provided clearly without using or mentioning the reference electrode [46,47], despite often stating that a reference electrode is required for a reproducible and stable signal from BioFET sensors [11]. This also raises a debate on the necessity of using a reference electrode in Si NWFETs. Consequently, the scarce information is very dispersed throughout the published papers.

Here, for the first time, beyond each particular case in the literature and without being completely exhaustive, this review attempts to list and report the various methods of employing Ag/AgCl reference electrodes in Si NWFET-based sensors for pH and biomolecule detection. The aim is essentially to classify them by providing a general overview of the different existing configurations through their implementation, while also reporting on the advantages and disadvantages of the variously encountered strategies. For instance, the methods of integrating Ag/AgCl reference electrodes will not be the same in the case of Si NWFETs equipped with either a microfluidic channel or a well. Moreover, some examples from both thin-Si film ISFETs and electrochemical sensors that could be transposed in Si NWFETS are also reported.

This review is divided into two main sections. The first and largest section (Section 2) describes the various kinds of Ag/AgCl reference electrodes used in Si NWFETs. Two categories of electrodes are distinguished depending on whether they are immersed or not in a saturated KCl salt solution. The saturated KCl solution provides the great advantage of keeping the electrode at a highly stable potential, which is very important for the needed application. The first category of electrodes gathers examples where either a standard commercial Ag/AgCl reference electrode is immersed in a saturated liquid KCl or is in contact with a solid KCl. Two subdivisions can be made according to the configuration of tested analytes: (i) in a microfluidic channel for dynamic measurements or (ii) in a well for static measurements. Nevertheless, the integration of such whole reference electrodes with KCl still remains challenging, especially within a microfluidic design or in the case of portable sensors. For this reason, some teams prefer working with a Ag/AgCl pseudo-reference electrode, i.e., without KCl solution. These electrodes are gathered in a second category. Unlike the electrodes described in the first category, these electrodes are home-made and are not commercially available. We will describe the different techniques used for their fabrication.

In the second section (Section 3), we face the question of different biasing possibilities of Si NWFET devices using Ag/AgCl reference electrodes. Their effects on some Si NWFET characteristics, such as sensitivity and noise, are reported. It is to be noted that any comparative work on the performance of Si NWFETs is a difficult task. Indeed, beyond the method of using Ag/AgCl reference electrodes, it is well known that numerous parameters are involved. These include the Si NWFET characteristics (architecture and design, top oxide layer, etc.), the bioreceptor layer (nature, conformation, surface density, etc.) [11,35], and the electrolyte characteristics (chemical nature, charge screening effect, and ionic strength) [48,49]. For these reasons, we limited our comparative study to a reporting of results of some papers.

## 2. Various Kinds of Ag/AgCl Reference Electrodes in Si NWFETs

Reference electrodes used in electrochemical measurement laboratories include calomel (Hg/Hg_2_Cl_2_) electrodes—referred to as saturated calomel (SCE) reference electrodes—standard hydrogen electrodes (SHEs), and Ag/AgCl electrodes. The first two types have proven to be very challenging to integrate on-chip. Ag/AgCl reference electrodes, being the easiest to miniaturize [50], have been the object of extensive research over the past few years, starting from external standard Ag/AgCl reference electrodes and moving up to integrated planar Ag/AgCl pseudo-reference electrodes.

### 2.1. External Standard Ag/AgCl Reference Electrodes

**Figure 2:**
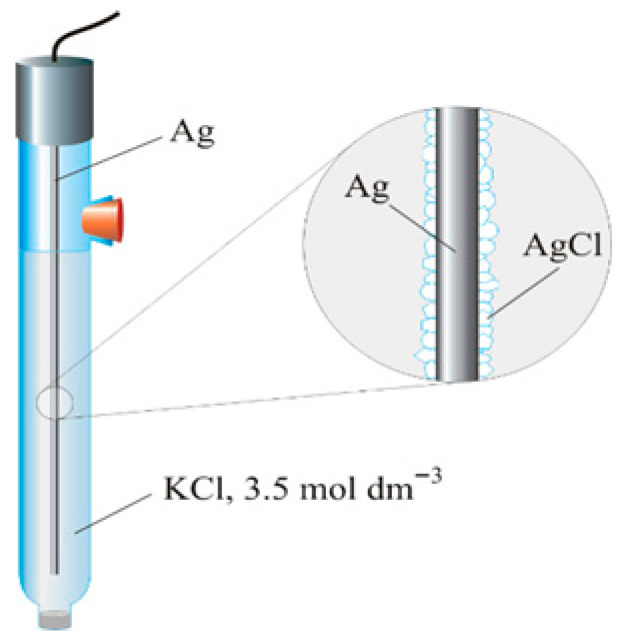
Structure of a conventional Ag/AgCl reference electrode in a KCl saturated solution [51].

Prior to listing the various Ag/AgCl reference electrodes used in Si NWFETs, let us review the operating principle of conventional standard Ag/AgCl electrodes. They consist of a chlorinated silver wire, dipped in a saturated KCl solution (typically in the order of 3 M), with a porous junction allowing ions to be exchanged between the analyte and the electrode (Figure 2). This junction allows the two solutions (KCl and analyte) to be separated, thus preventing the contamination of the analyte with KCl ions [51]. The operation of the Ag/AgCl reference electrode relies on the following reaction:

AgCl_(s)_ + e^−^ ⇄ Ag_(s)_ + Cl^−^_(aq)_

where (s) refers to the solid state and (aq) refers to the solvated state. This provides an electrochemically stable interface with the reference electrode via a well-defined redox reaction between the silver metal (Ag)_S_ and its salt (AgCl)_S_. The highly concentrated KCl solution ensures the minimal dependency of the electrode from eventual chloride concentration changes in the test solution, and is usually in contact with this solution via an ion conductor bridge [52]. When using Si ISFETs for biosensing, the Vth shift obtained via pH variation or charged biomolecule detection depends on the change in the surface potential only when the potential at the reference electrode is stable [52], as described above. In view of their use and integration in miniaturized biosensors, manufacturers have made efforts to commercialize standard Ag/AgCl reference electrodes with reduced dimensions. For example, World Precision Instruments (USA) develops electrodes with a diameter of 450 µm with an extremely low electrolyte leakage.

Si NWFET biosensors can be designed differently depending on the configuration of the electrolyte, which can be in either static or dynamic conditions. In the first case, a small drop of liquid is deposited on the sensing circuit, allowing only static measurements; in the second case, a microfluidic channel is located on the sensing NWs, enabling liquid circulation and thus real-time measurement. Depending on the liquid configuration, the positioning of the standard Ag/AgCl reference electrode varies, being much more challenging in the case of a microfluidic circulation. In the following subsections, we report some examples from the literature where the external standard Ag/AgCl reference electrode is first used in microfluidic Si NWFET systems and then in static systems.

#### 2.1.1. Case of Si NWFET Devices with a Microfluidic Channel

**Figure 3:**
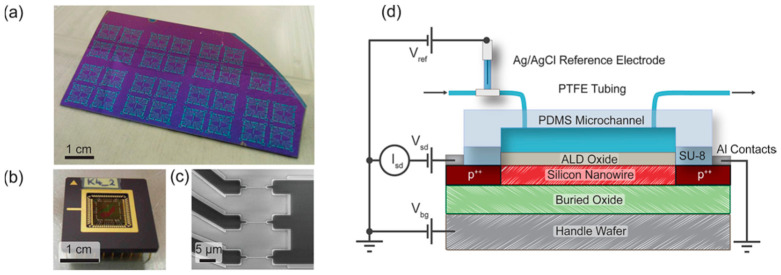
(**a**) Optical image of a wafer piece after electron beam lithography of the nanowires. The samples are arranged in 2 × 2 squares. Each sample consists of 48 nanowire FETs. Light areas are the nanowires and contact leads, while dark areas are the buried oxide. (**b**) Sample after bonding into a chip carrier. (**c**) SEM image of three nanowires (thin horizontal lines) with contact areas on the left (source) and a common bus line on the right (drain). (**d**) Cross-section of the fabricated device and a sketch of the measurement setup (not to scale). The liquid is delivered to the custom-made PDMS microchannels by a pump (indicated by arrows). A flow-through Ag/AgCl reference electrode is integrated in the Teflon (PTFE) tubing close to the microchannel. The working point of the nanowire transistor is adjusted by two voltages: a back-gate voltage Vbg (applied to the handle wafer) and a liquid-gate voltage Vref (applied to the reference electrode). Reprinted with permission from Reference [49]. Copyright © 2012 American Chemical Society.

Reference electrodes can be positioned either outside or inside the microfluidic channel, where measurements are conducted. In the first case, like in the study by D. Kwon et al. on the drain current (ID) drift of Si NW ISFET-based pH sensors [53], the RE is placed in an outside waste container, meaning far away from the sensing surface (NW array). In the second case, the RE is placed closer to the sensing NWs, being directly at a junction in the liquid inlet tube, as illustrated in Figure 3 [49].

**Figure 4:**
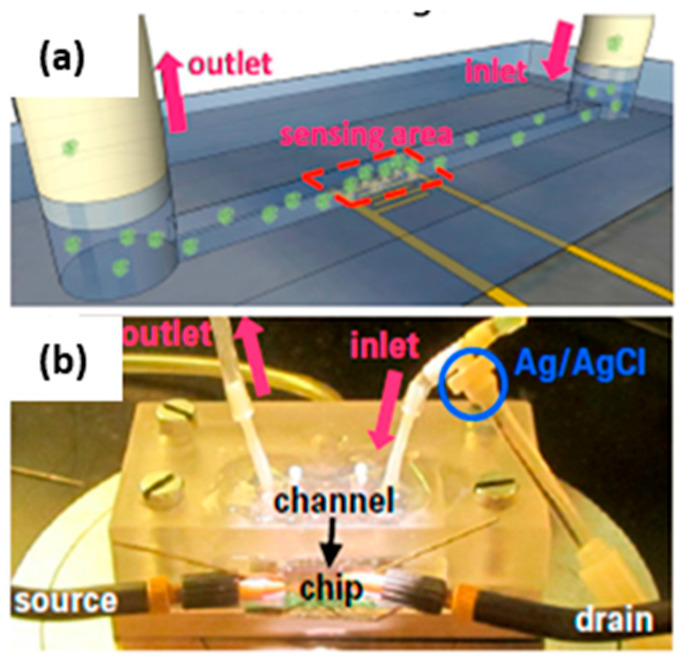
(**a**) Sensor concept with fluidic channel above nanowire region and realization of inlet and outlet flow. (**b**) Real fluid cell setup with contacted source and drain and PDMS channel for sample delivery as labeled, and position of the Ag/AgCl reference electrode. Microchips are of approximately 2 × 2 cm^2^. Reprinted from Ref. [54]. Available under CC BY 4.0 license.

This second configuration is also the one chosen in the work of both L. Römhildt et al. [54] and B. Ibarlucea et al. [55], who implemented a Si NWFET biosensor for the highly specific and sensitive instantaneous detection of human alpha thrombin. The active sensor surface is covered with a 15 nm thick Al_2_O_3_ gate oxide, which is functionalized with thrombin-binding aptamers as receptor molecules. A leak-free Ag/AgCl reference electrode (purchased at Microelectrodes Inc., Bedford, NH, USA) installed at the inlet tube assures measurement against a stable liquid potential (Figure 4a,b) [54].

Elsewhere, for pH measurements, S. Rigante et al. [56] proposed FinFETs made from SOI wafers, featuring a HfO_2_ gate oxide architecture for optimized channel control, sensitivity, and stability. Such devices are known to exhibit excellent electronic properties, which are well known in nanoelectronics [56]. The liquid potential is controlled by a Ag/AgCl flow through the reference electrode included in the tubing. High long-term stability was proven over 4.5 days, with a drift in time limited to 0.14 mV/h [56].

For similar application of pH measurements as a function of time, Si NWFETs were fabricated by G. Lehoucq et al. [57] on Si NWFETs, without specific mention of the gate oxide nature. The authors demonstrated a change in the transistor conductance according to the pH of the solution on a large pH interval [3–10.5], even for small variations of 0.1 pH units. The influence of several physico-chemical parameters, such as the gate voltage and buffer salinity, usually not adequately taken into account in previous papers, is discussed to achieve a better understanding of the detection phenomena [57]. The description of the location of the Ag/AgCl reference electrode is not clearly presented in the paper but is rather specified in the corresponding Ph.D. work [58]. Thus, a non-miniaturized Ag/AgCl reference electrode was placed in a container filled with electrolyte, into which the outlet capillary was immersed. This configuration seems to be similar to that of the previously cited study of D. Kwon et al. [53].

The configuration in which the reference electrode is connected directly to the microfluidic channel offers the great advantage of positioning the electrodes fairly closer to the sensing area than when the electrode is placed in a waste container. However, combining a conventional reference electrode with a microfluidic setup can be challenging. A simpler method consists of using a reference electrode dipped in a liquid drop contained in a well, which is positioned over the sensing area. However, this static configuration does not allow for dynamic real-time measurement but it provides a useful way to conduct first-step analyses regarding the behavior of the biosensing area containing nanowires, and to be sure of the characteristics in liquid conditions. Some examples of these static configurations are listed in the following section.

#### 2.1.2. Case of Si NWFET with a Well for Static Liquid Analyses

**Figure 5:**
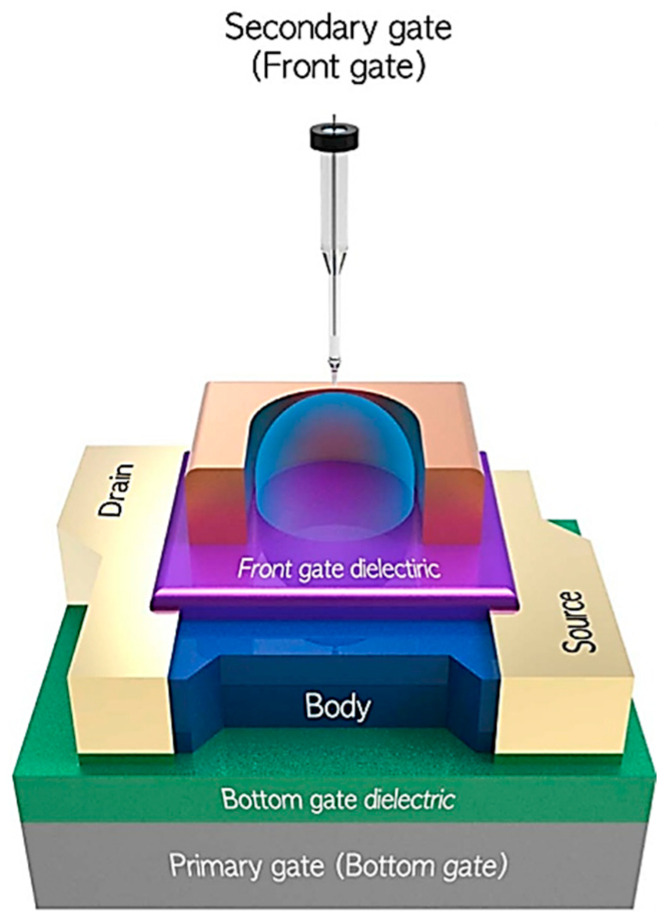
A cross-sectional schematic image of a dual-gate (DG) ISFET. The designed active channel length and width are 10 µm and 10 µm, respectively. The thicknesses of the top active Si layer and of the BOx are 4.3 nm and 224 nm, respectively. A top SiO_2_ layer with a thickness of 23 nm is grown by thermal oxidation for the sensing membrane. A commercial Ag/AgCl reference electrode is used for various pH buffer solutions. Reprinted from Ref. [61]. Available under CC BY 4.0 license.

Commercial Ag/AgCl standard electrodes were first historically used in the case of ISFETs for static liquid analyses. For instance, they were used with Ta_2_O_5_-gated ISFETs for the aptamer-driven detection of protein (troponin I) [59]. They were also used in the case of high-performance dual-gate (DG) ISFETs—as will be described further (Section 3)—for instance, in the case of thin-Si-film transistor-based ISFETs used by T. E. Bae et al. [60] and H. J. Jang et al. [61]. A cross-sectional image of their dual-gate ISFET device with an external Ag/AgCl electrode is presented in Figure 5 [61]. The authors have provided a comprehensive study of the effect of the body (Si channel film) thickness on the pH sensitivity. As a result, an ultra-thin body (UTB) of 4.3 nm serves not only to increase the sensitivity but also to strongly suppress the leakage components, leading to the better stability of the DG ISFET. A thicker body can lead to a degradation in the device performance, affecting key parameters such as sensitivity and stability (see Section 3).

Elsewhere, J. W. Kang et al. [62] used a standard reference Ag/AgCl electrode (Horiba 2080A-06T with a ceramic plug junction and an internal solution saturated with KCl and AgCl to measure the pH sensitivity on an EGFET (extended-gate FET). This type of device is a derivative of the ISFET that separates the sensing domain (the extended gate) from the transistor’s gate, connecting the two with a conductive wire. This configuration allows the transistor to remain in a dry environment, while the sensing domain interacts with the analyte in a wet environment [63]. Thus, the authors [62] could compare the pH sensitivity depending on the kind of sensing top oxide/gate insulator (membrane) deposited on the extended gate (see further in Section 3).

**Figure 6:**
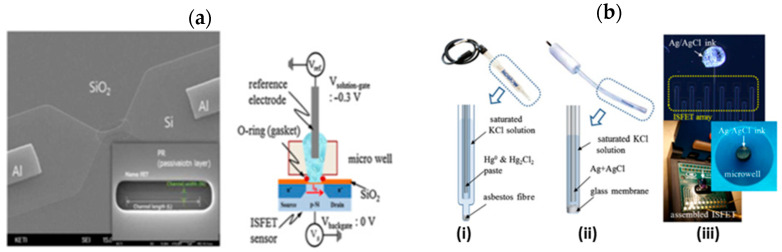
(**a**) FESEM image of a Si NWFET with a microwell (**left**), and schematics for measuring the readout current for stabilization of the sample solution potential with a reference electrode (**right**). (**b**) Use of three reference electrodes for comparative study: (**i**) SCE-saturated calomel reference electrode, (**ii**) Ag/AgCl reference electrode in saturated KCl solution tube, and (**iii**) integrated reference electrode on chip with Ag/AgCl ink. The dimensions are a 5 nm thick top oxide and a 20 nm thick channel Si NW on a 145 nm thick BOx layer. The length channel is 2 µm. The chip size is about 5 × 7 mm. Reprinted from Ref. [64]. Available under CC BY 4.0 license.

In the case of Si NWFETs for static liquid analyses, commercial standard Ag/AgCl electrodes are also used. For instance, Y. T. Seo et al. [64] used a refillable miniature Ag/AgCl reference electrode to evaluate the pH and ion concentration sensitivity in an open reservoir configuration or in a micro-well built on a nanoFET device fabricated on an SOI wafer (Figure 6a). The active Si channel and insulator layer (BOx) were 20 and 145 nm thick, respectively. The top gate oxide was 5 nm thick. Moreover, being aware of the crucial role of the reference electrode in sensing measurements, the authors focused their study on the effect of the reference electrode on the signal. They evaluated how the use of a refillable Ag/AgCl reference electrode affected the sensor sensitivity in comparison with both a saturated calomel reference electrode (SCE) positioned in the microwell and with an integrated Ag/AgCl ink (eDAQ Co., Ltd., Nagoya, Japan) obtained by printing deposition on a chip (Figure 6b). This will be reported further (Section 3).

**Figure 7:**
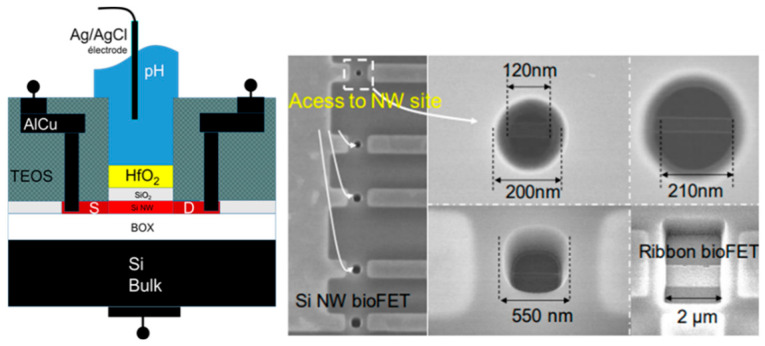
(**left**) Cross-sectional schematic of Si NWFETs with standard commercial Ag/AgCl reference electrode, and SEM images (**right**) showing ultra-scaled opening access with diameter dimensions ranging from 250 nm down to 120 nm on top of the Si NW array. The latter is made up of 15 Si NWs, each with a 50 nm width. Reprinted from Ref. [65]. Available under CC BY 4.0 license.

R. Midahuen et al. [65,66] have reported a wafer-scale fabrication of biologically sensitive Si NWFETs with various Si nanostructure architectures (nanowire array, nanoribbons, and honeycomb) for static pH measurements, DNA hybridization, and thrombin detection. A high-quality 3 nm thick SiO_2_ layer was thermally oxidized on the Si surface before the deposition of a 4 nm thick HfO_2_ layer. The latter was selected because of its excellent pH sensing properties and its chemical inertness in most acid and basic solutions. A standard commercial Ag/AgCl reference electrode was used (Micro-Dri-Ref, 450 µm diameter from World Precision Instruments). A variety of access points, with dimensions ranging from 250 to 120 nm, were provided for the liquid to reach the sensing NW sites, the dimensions of which are described in Figure 7. A cavity diameter limit was found at 210 nm, under which no sensor was functional due to the wettability limit inside the too-small open cavity. Moreover, in certain experimental conditions involving both the reference and the back-gate electrodes, an enhancement of the FET sensitivity to pH could be obtained thanks to a capacitive coupling between the reference and the back-gate electrodes. This will be detailed further in Section 3.

All of the sensing devices described above used a commercially available, miniaturized, and leak-proof Ag/AgCl reference electrode. We have observed that, from one publication to another, some information is missing. The full range of parameters necessary for detection measurements, such as the device’s geometric and electrical characteristics, electrolyte characteristics, and polarization conditions, are rarely reported simultaneously. Consequently, a rigorous and objective comparative analysis of device performance (sensitivity, temporal stability, etc.) proves difficult. To facilitate such comparative studies in the future, we have listed (Table 1) all of the parameters to be considered and included in any publication.

As an alternative to using a standard external Ag/AgCl reference electrode, which requires no additional manufacturing or on-chip integration steps, we present, in the following subsections, several examples of devices where the Ag/AgCl reference electrode is fully integrated onto the chip. This approach requires an additional manufacturing step that must be taken into account.

### 2.2. Fully Integrated Ag/AgCl Electrode with KCl

As an alternative to the commercialized standard external Ag/AgCl reference electrode (as described in Section 2.1.1), attempts have been made to entirely integrate a whole Ag/AgCl reference electrode in FET devices. This includes the integration of a KCl solution, which is of utmost importance to keep the Ag/AgCl reference electrode at a very stable potential. The KCl can be in either a liquid or solid state.

#### 2.2.1. Fully Integrated Ag/AgCl Electrode with Liquid KCl

**Figure 8:**
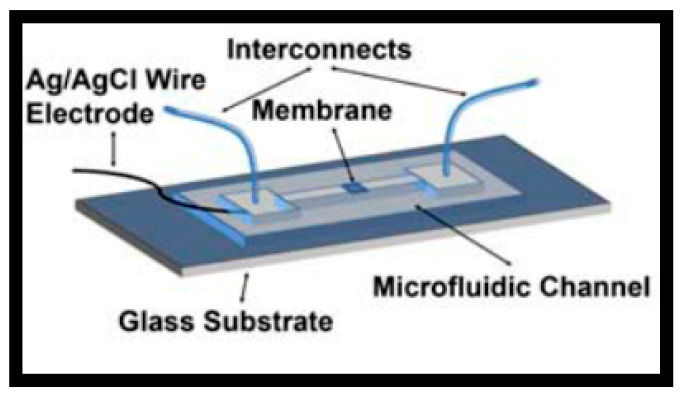
Schematic of a whole miniaturized micro-reference electrode (µF). A Ag/AgCl wire electrode is connected to a microfluidic KCl channel (3 cm × 500 µm × 80 µm) embedded in PDMS. Reproduced from Refs. [67,68] with permission from the Chemical and Biological Microsystems Society (CBMS). Copyright (2010) CBMS.

This kind of electrode has been particularly described in the context of electrochemical potentiometric sensor chips using porous junctions, such as polymers, porous glass, porous silicon, or other porous materials, which are listed in [50]. It is crucial to have a leak-free junction between the different solutions to avoid the leakage of the KCl solution into the analyte, which would lead to the contamination and/or destruction of the sample. For general applications in the BioFET sensor system, S. Safari-Mohsenabad et al. [67,68] built a whole miniaturized microfluidic Ag/AgCl reference electrode (µF reference electrode) consisting of a Ag/AgCl wire electrode connected to a microfluidic KCl channel (3 cm × 500 µm × 80 µm) embedded in PDMS, as illustrated in Figure 8. A free-diffusion liquid junction was established through a polycarbonate membrane (100 nm pores). The potential stability and lifetime of this microfluidic reference electrode were characterized against a macroscale commercial Ag/AgCl reference electrode in various modes of operation to determine the optimal configuration, notably by observing the effects of important parameters such as the internal solution flow, pH, and concentration of Cl^−^ of the external solution, such as PBS electrolyte. Results showed a stable potential (<40 µV) and a lifetime of at least 100 h. Nevertheless, the presence of liquid KCl salts can induce leakage, thereby damaging the devices. For this reason, attempts have been made to employ a solidified phase of KCl salts, as described in the following subsection.

#### 2.2.2. Planar Solidified Ag/AgCl Electrode Filled with Solid KCl

To avoid the leakage of the reference solution from the internal liquid KCl solution in the sensing devices, various kinds of potentiometric electrochemical chip sensors are instead based on planar, fully solidified Ag/AgCl reference electrodes, thus prolonging the lifetime of the system. As early as 2009, U. Guth et al. [69] reviewed the different development principles of Ag/AgCl reference electrodes, with particular attention to the KCl electrolyte characteristics. If gel-based reference electrodes—in which KCl salts are dissolved—were first used, the evolution moved towards the insertion of salts into strong hardening polymers, such as polyvinyl chloride, polyethylene, and epoxy resins and vinyl esters [70]. In the case of ISFETs, the use of a conventional liquid-filled Ag/AgCl reference electrode, even when drastically miniaturized, would have considerably impaired the attractiveness of ISFETs as all-solid, small-sized microelectronic sensor devices. Like electrochemical biosensors, ISFETs have also benefited from these new kinds of solid-state reference electrodes. Considerable efforts have been made to develop adequate solid reference electrodes for ISFET sensors, as described below.

#### 2.2.3. Other Solid-State Reference Electrode Configuration

As reviewed by U. Guth et al. [69], the creation of reference ISFETs (REFETs) has been performed with the implementation of a modified pH-insensitive solid membrane on the surface of the dielectric gate of the ISFET. This modified ISFET can be combined with a standard ISFET and a counter electrode (usually a Pt electrode wire). This means that two half-cells are connected, i.e., an ISFET without any reference electrode and a FET with a reference (REFET) [70,71]. Such systems share a common electrode between two transistors, enabling pH detection in differential mode measurements while remaining compatible with iC-CMOS technology [72]. The inherent advantages of a differential measurements are, namely, temperature and noise compensation [72,73]. K-M. Chang et al. [74] described an immobilized urease enzyme layer (like Nafion^TM^) coated on the surface of ISFET gate dielectrics used in combination with a noble metal electrode (Pt), serving as a quasi-reference electrode (QRE), and integrated with an ISFET (QRE/ISFET/REFET) associated with two-ended differential readout circuits. This provides a wide dynamic operating measurement range, achieving similar urea response curves as those measured by conventional large-sized discrete sensors. These developments were key points for larger-scale integration still aiming to avoid the presence of liquid with the reference electrode. More recently, G.T. Ayele et al. developed CMOS-compatible pH sensors in the back-end-of-line (BEOL) of industrial 28 nm ultra-thin body and buried oxide (UTBBB) fully depleted silicon-on-insulator (FDSOI) transistors [75]. The sensing gate and the control gate were fabricated in a capacitive divider circuit in such a way that the front-gate bias was applied through the control gate rather than a bulky reference electrode.

As mentioned, the KCl solution, either in liquid or in solid state, gives the great advantage of keeping the reference electrode at a very stable potential, which is crucial for the application needed. However, the full integration of such a whole Ag/AgCl reference electrode can be difficult and challenging within a microfluidic design, even if miniaturized reference electrodes are currently developed and commercialized in order to suit small sensing devices, as described above. That is why, in order to reduce difficulties, many teams prefer to use a pseudo-reference electrode without using any KCl salts, as suggested by J. Janata et al. [73].

### 2.3. Pseudo- or Quasi-Ag/AgCl Reference Electrode

A pseudo-reference or quasi-reference electrode (QRE) can be either a bare metal sample or layer (Pt, Au, or Ag), or a chlorinated silver wire without any KCl solution, dipped directly in the electrolyte (analyte). The aim with such electrodes is to provide a fairly stable potential, which is not known a priori [40,41]. Unfortunately, this potential can change with the salt composition (Cl^−^) of the solution or with the pH, which can be a problem when detecting biomolecules. Therefore, the choice of the metal is crucial so to have a stable enough electrode with which to conduct the measurements. N. K. Rajan et al. demonstrated that the use of a Ag/AgCl wire was the best choice in terms of pseudo-reference electrodes to conduct biosensing measurements [40,41]. Indeed, bare wires of Pt and Au have a tendency to be highly dependent on pH and more prone to noise than Ag/AgCl wires. However, it was also demonstrated that the Ag/AgCl wire could provide a stable enough potential to be a pseudo-reference electrode only if the concentration of Cl^−^ ions was kept constant in the analyte. Indeed, a fluctuation in Cl^−^ will cause a redox reaction at the AgCl/electrolyte interface, therefore changing its potential and skewing the results. In the following, we describe the two main geometry configurations of Ag/AgCl pseudo-electrodes, i.e., wire or planar. They rely on different Si NWFET fabrication processes.

#### 2.3.1. Ag/AgCl Pseudo-Reference Electrode Wire

Ag/AgCl pseudo-reference electrode wires are simply silver wires which have been chlorinated [67,76,77]. To obtain a uniform and hardly soluble AgCl salt layer on the Ag wire, it is first necessary to clean the silver wire or silver tubing with ethanol so to remove any contaminants. Then, to chlorinate a silver wire, two techniques can be used: chemical oxidation or electroplating. Chemical oxidation is obtained by immersing the wire in household bleach for 15 to 30 min until a purple/gray color is observed on the wire. When using electroplating, the wire is immersed in a NaCl (0.9%) or KCl (3 mM) solution and a current passes through the wire at a rate of 1 mA/cm^2^ for 10 to 15 s [51].

**Figure 9:**
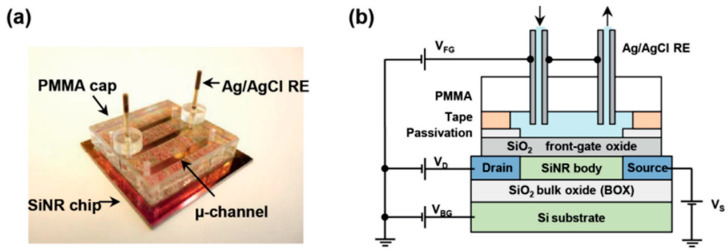
Chip that integrates pseudo-Ag/AgCl REs, microfluidics, and sensing nanodevices: (**a**) optical image of the mounted microfluidics setup; (**b**) cartoon showing the cross-section of the Si Nanoribbon (NR)-ISFET coupled with the microfluidic module (not to scale). From bottom to top: (i) Si Nanoribbon (NR) chip; (ii) double-coated tape (the height of the channels is defined by the thickness of the tape, at 190 μm); (iii) PMMA cap as sealing polymer with the Ag/AgCl inlet and outlet tubes to avoid fluid leakages from the inlets/outlets [15]. Reprinted with the permission of the authors.

**Figure 10:**
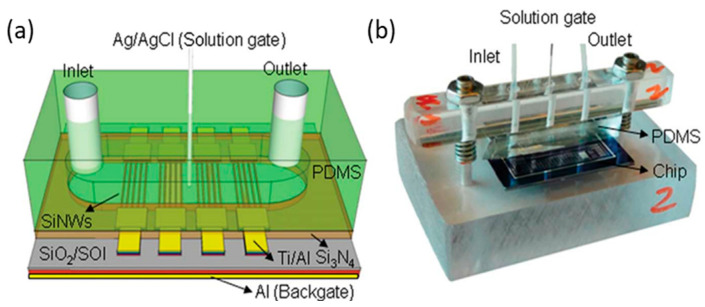
(**a**) Schematic diagram of a multi-SiNW bioFETs with an integrated PDMS fluidic channel, Ag/AgCl wire as a solution gate, and Si substrate as a back-gate. (**b**) Image of an assembled multi-SiNW FET sensing platform. Reprinted with permission from Reference [76]. Copyright © 2011 Royal Society of Chemistry.

An original design was proposed by E. Accastelli et al. [15,78,79]. They employed chlorinated silver tubing to flow the analyte through, so that only the inside of the tubing has been chlorinated. These tubes are inserted into a microfluidic chip, serving both as REs and as inlet/outlet of the system in such a way that the electrode is completely integrated in the microfluidic circuit (Figure 9). This is a simple and reliable way to solve the issue of the integration of the pseudo-reference electrode within a biochemical sensing platform. The fabrication process of this pseudo-reference tube electrode involves flowing a 0.1 M KCl solution through the tubing while passing an anodization current at a rate of 2 mA/cm^2^ through the tube, resulting in a AgCl layer thickness of about 5 µm. The performance of these pseudo-reference tube electrodes were compared with respect to commercial Ag/AgCl electrodes [79]. An important point is that only the AgCl layer of the Ag wire (or tube) is exposed to the liquid [67]. Elsewhere, in view of ultrasensitive bovine serum albumin (BSA) protein detection, Tian et al. [76] built a polydimethylsiloxane (PDMS) micro-machined fluidic channel with aligned inlet/outlet ports. This setup was brought into contact with the Si NW chip and sealed using a mechanical clamp. A metal wire (Ag/AgCl) was inserted through the PDMS into the solution, serving as the solution gate (Figure 10a,b).

Using silver wires or silver tubing as a pseudo-reference electrode offers one main advantage. In the case of a degraded reference electrode, this setup makes it easy to replace the electrode with a new one [80]. However, this strategy is not compatible with the miniaturization of the circuit and can be difficult to integrate on-chip. Recognizing these limitations, some works report the use of planar pseudo-reference electrodes.

#### 2.3.2. Planar Ag/AgCl Pseudo-Reference Electrodes

In the case of planar configurations, the Ag/AgCl layer is directly embedded on the chip containing the Si nanowires. Thus, its fabrication must be integrated during the Si NWFET chip fabrication process.

**Figure 11:**
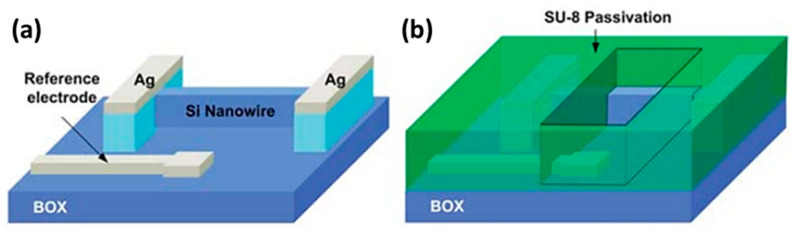
Planar pseudo-reference electrode integrated on the Si NW-based ISFET chip: (**a**) after the Ag/AgCl layer deposition; (**b**) after the 2 µm thick SU-8 layer passivation on the chip, except for the active sensing region and the contact pads, to prevent leakage current between the contacts and the solution. The BOx thickness is 200 nm. The Si NW has a thickness of 40 nm thick, width of 50 nm, and length of 10 µm. The gate oxide is 5 nm thick. The width, length, and height of the well holding the solution over the devices are 1200 µm, 1200 µm, and 2 µm, respectively. The width and length of the pseudo-Ag/AgCl reference electrode are 1000 µm and 1000 µm, respectively (thickness not described). Reprinted with permission from Reference [80]. Copyright © 2011 Royal Society of Chemistry.

The fabrication process involves the deposition of a Ag layer onto the silicon substrate, which is then chlorinated to form a AgCl layer on top of the existing Ag layer. Several methods are described to form this AgCl layer. An example of the on-chip integrated planar pseudo-reference Ag/AgCl electrode type is illustrated in Figure 11 [80]. The authors fabricated Si NW-based ISFETs in view of biosensing applications, emphasizing the pH sensing and noise characteristics. Following the fabrication process steps of Si NWs covered with the 5 nm thick gate oxide, the evaporation and patterning of the source/drain metal contact pads as well as the gate (reference) electrode (20 nm thick Ti and 200 mn thick Ag layers) were performed using conventional lithography and a lift-off process. Then, they used the electrochemical deposition of AgCl particles on top of the Ag layer. They dropped 30 µL of 0.1 M KCl solution onto the silver film and applied a voltage of 1 V for 300 s. This resulted in the deposition of a AgCl layer due to the reaction between silver and chloride ions, forming a Ag/AgCl electrode of 1000 µm in width and 1000 µm in length (thickness not described). Finally, the surface of the entire device, except for the active sensing region and the contact pads, was covered with a 2 µm thick SU-8 layer to prevent leakage current between the contacts and the solution. By sweeping the drain current as a function of the Ag/AgCl gate voltage between 0 and 1.5 V, with a grounded substrate to increase the pH values, the pH sensitivity was found to be around 40 mV/pH, which was estimated to be a typical value for ISFETs using the SiO_2_ gate insulator as a sensing layer [80]. K. Kim et al. [81] developed a Si honey comb nanowire (HCNW)-based biosensor for the detection of cardiac troponin I (cTnI). To fabricate the embedded pseudo-reference Ag/AgCl electrode, the authors used a similar fabrication process as that previously described [80]. Regarding the stability and lifetime of fabricated Ag/AgCl layer gates, no specific information was provided by either study. However, due to the small size of the electrode, the AgCl layer is often deposited with a small thickness (a few hundred nanometers). Unfortunately, with such dimensions, the AgCl layer can be dissolved rather quickly, leading to time stability problems and the reduced lifetime of the electrode [15,35,36]. This problematic is taken into account in the following studies that aim to optimize the deposition process of the Ag/AgCl electrode layer.

B. J. Polk and coworkers [82] constructed micrometer-sized quasi-reference electrodes and electrode arrays which can be used in microfluidic applications for electrochemical analyses. They discussed the practical aspects of the fabrication and characterization of stable Ag/AgCl microelectrodes, both individually and in an array format. Following the Ag metal evaporation deposition, they tried to chlorinate the Ag layer using both electrochemical anodization in HCl and chemical oxidation with aqueous FeCl_3_. They found the chemical oxidation to be the most efficient, providing a uniform layer in the smallest amount of time. A solution of 50 mM FeCl_3_ was deposited on the silver layer for 50 s before being rinsed with deionized water. The importance of performing the oxidation step of the silver film just after being deposited was also noted, thus preventing the formation of silver oxide or silver sulfide, which would impair the quality of the AgCl layer. Finally, by replacing the Ag evaporation by Ag electroplating, which provided a thicker and rougher Ag film, the problem of rapid dissolution could partially be avoided. Indeed, this deposition technique enables the deposit of a larger quantity of silver on the AgCl layer [82]. Using this method, the authors could obtain an electrode stability of 4 days [82].

In his Ph.D. thesis, N.K. Rajan [41] provided detailed work comparing the efficiency of two kinds of pseudo-reference electrodes, including Pt metal and planar Ag/AgCl pseudo-electrode on Si NWFETs versus a standard Ag/AgCl reference electrode. He also compared two methods to obtain a planar Ag/AgCl pseudo-electrode. First, the electrochemical oxidation of the e-beam evaporated Ag layer was tested by applying 3 V to the silver during 15 min, using a Pt electrode as ground. However, this method of AgCl deposition does not give a lot of control over the quality and thickness of the layer deposited. That is why chemical oxidation was used instead. The deposition process performed by immersing the Ag layer in 10% household bleach is slower, thus providing a better control over the layer. The resulting electrodes were examined both optically and by considering changes in the open-circuit potential. This Ag/AgCl layer was found to result in a fairly stable potential over time (several hours), with a relatively small interfacial potential (30–60 mV w.r.t Ag/AgCl reference). Additionally, these results highlighted the potential stability issues of the Pt electrode compared to the Ag/AgCl pseudo-reference electrode, which was found to be as robust as the full reference electrode. The author also ensured that the on-chip Ag/AgCl electrode maintained a constant interfacial potential, even as the pH of the solution was changed. However, the chloride concentration in the solution should still be carefully controlled. Finally, in situations where the limit of detection (LOD) of the setup becomes an important consideration, such as sensing small analyte concentrations under low-quality conditions, a full reference electrode still yields a higher peak signal to ratio, and therefore a smaller LOD, than either one of the pseudo-reference electrodes.

**Figure 12:**
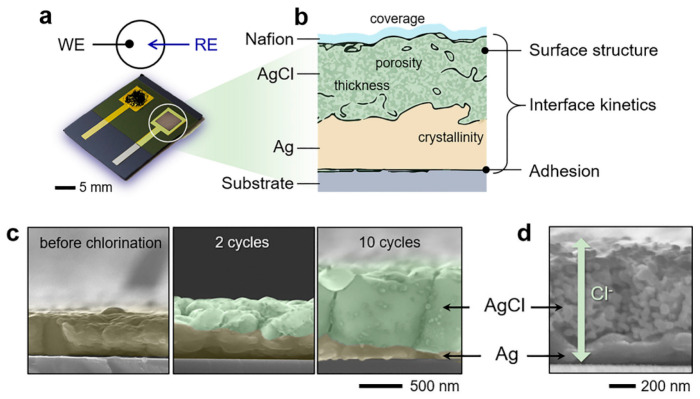
Electrochemical sensor structure and characterization of a Ag/AgCl/Nafion reference electrode (RE). (**a**) Photo of a miniaturized, two-electrode setup, including a working electrode (WE) and an RE. (**b**) Schematic illustration of the cross-sectional view of the structural interface of the newly fabricated, all-solid-state Ag/AgCl/Nafion. (**c**,**d**) Cross-sectional SEM images that capture the layered structure of Ag/AgCl electrodes formed up to 10 cyclic voltammetry cycles (**c**) and the porous structure of primary AgCl nanoparticles (**d**). Reprinted with permission from Reference [83]. Copyright © 2020 Elsevier.

In view of improving the robustness of the planar solid-state Ag/AgCl pseudo-reference electrodes, numerous efforts have been performed in the field of electrochemical sensors by optimizing the fabrication process, and notably the chlorination factors. This has been thoroughly described by H.-R. Lim et al. [83] for devices designed for the electrochemical monitoring of glucose. According to these authors, ensuring the long-term stability of a thin-film RE requires a conformal coating of AgCl without any structural instabilities, which can induce delamination. After the e-beam evaporation of Ag on Ti covered Si wafer, followed by a vacuum annealing to improve adhesion and cooling under nitrogen to room temperature to minimize surface oxidation, a thorough process of chlorination was performed using a cyclic voltammetry strategy. An illustration of the cross-sectional view of the structural interface of the newly fabricated, all-solid-state RE is reported in Figure 12. This allowed for an easy, low-cost, rapid control of the Ag/AgCl film thickness and conformal coating of the AgCl layer, offering an 800 nm thick, uniform RE surface with improved adhesion and a smooth surface AgCl film (<50 nm roughness). Finally, the electrode was coated with a Nafion film as a protective layer. As a result, the long-term stability of the solid-state electrode over two weeks was obtained.

**Figure 13:**
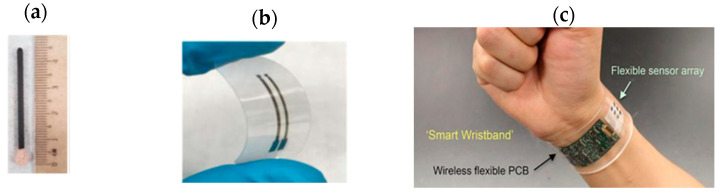
(**a**) Schematic representation illustrating a planar Ag/AgCl pseudo-reference electrode obtained by 3D printing. Reprinted from Reference [85], available under CC BY-NC-ND license. (**b**) Photo of the first fabricated full-ink-jet-printed solid-state reference electrode on a 125 µm thick polymeric (polyethylene terephthalate–PET) film substrate. Reprinted with permission from Reference [84], Copyright © 2019 American Chemical Society. (**c**) Photo of a wearable flexible integrated sensing array (FISA) on a subject’s wrist, integrating the multiplexed sweat electrochemical sensor array and the wireless flexible printed circuit board (FPCB). Reproduced from [86] with permission from the SNCSC.

More recently, to overcome the issues of the lifetime and stability of the planar pseudo-reference electrode, printing techniques have emerged as standard methods for the mass production of low-cost and rapid prototyping of miniaturized electrodes [84]. A straightforward, fast, and simple method was used by N. Rohaizad et al. [85] to prepare macroscopic Ag/AgCl pseudo-reference electrodes (Figure 13a) from 3D-printed graphene/polylactic acid filaments, which are extremely cost-effective and widely available [85]. The fabrication process involves the optimized electrodeposition of Ag followed by bleaching to form AgCl on the surface of this electrode. The electrodeposition and bleaching parameters, such as time, were optimized based on the open-circuit potential measurements against commercial reference electrodes. However, the time stability of the obtained electrodes is not mentioned. Elsewhere, A. Moya et al. [84] developed a stable and reusable full-inkjet-printed solid-state reference electrode (IPRE). The latter was deposited on a flexible 125 µm thick polymeric (polyethylene terephthalate—PET) film substrate (Figure 13b) The Ag ink layer was first printed and then chlorinated by sodium hypochlorite NaClO printing. Finally, a coating was applied by printing a Cl^−^-saturated polyvinyl butyral (PVB) membrane. The latter has two roles: (i) to protect the outer surface of the reference electrode by forming a diffusion barrier that avoids the dissolution of the small volume electrode and increases the stability of the electrode, and (ii) to allow the ionic contact between the solution and the phase boundary acting as a liquid junction bridge. The stability of such reference electrodes was extensively explored in the two most common harsh conditions of Ag/AgCl-based reference electrodes: the pH and Cl^−^ concentration of the measuring solution. As a result, no relevant potential change was observed in a wide range of pH from 2 to 11, and the membrane was shown to not capture further ions, as the potential was stable over 10^−6^ to 10^−2^ M. These low-cost miniaturized and flexible reference electrodes are suitable for use in miniaturized electrochemical devices. Such Ag/AgCl reference electrodes were fabricated in view of being inserted and used in potentiometric sensors or complete electrochemical flow cells, opening a path to the development of wearable sensor arrays.

The integration of these planar solid-state pseudo-reference electrodes in wearable electrochemical sensors has been extensively studied. W. Gao et al. [86] fabricated a “smart wristband”. The wristband is a fully integrated wearable sensor array for multiplexed in situ perspiration analysis (Figure 13c), notably for the real-time electrochemical amperometric detection of metabolites, such as glucose, lactate, and ions (Na^+^ and K^+^). These enzymatic-based electrochemical sensors autonomously generate current signals proportional to the abundance of the corresponding metabolites between the working electrode and the Ag/AgCl electrode. The latter is 180 nm thick and 3 mm in diameter. It is obtained by Ag patterning followed by lift-off in acetone. The chlorination is obtained by injecting 10 µL of 0.1 M FeCl_3_ solution for 1 min on top of the Ag area.

The integration of these planar solid-state pseudo-reference Ag/AgCl electrodes in flexible and wearable ISFETs has been developed more recently. This was performed for the first time in 2017 by F. Bellando et al. [43] and continued a few years later [87,88]. They built ISFETs for wearable multi-sensing sweat applications, called Lab-on-Skin^TM^ [43]. The sensors are fabricated by wafer-level 3D heterogeneous integration of ion-sensitive fully depleted (FD) FETs with a biocompatible SU-8 micro/nanofluidic interface. The fully depleted SOI substrate provides excellent electrostatic control and low leakage current.

**Figure 14:**
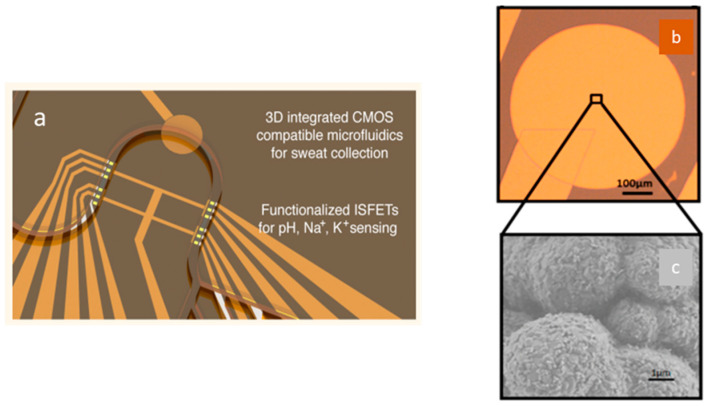
Three-dimensionally integrated microfluidics with ISFET for wearable sweat analyzer: (**a**) pseudo-color top view zoom on a microfluidic channel over Au gate ISFET and the circular quasi-reference electrode (QRE); (**b**) zoom on the miniaturized QRE; (**c**) SEM image of the chlorinated Ag surface. Reprinted with permission from [88]. Copyright © 2018, American Chemical Society.

The SOI FET sensors exhibit ribbon-like form factors with a Si film thickness of 30 nm and channel widths ranging from 0.8 to 4 µm. A 3 nm thick HfO_2_ gate dielectric offers nearly Nernstian sensitivity to pH and ultra-low gate leakage. Moreover, they report the integration of a miniaturized Ag/AgCl quasi-reference electrode (QRE) into the sensing system (Figure 14a,b). A 3 µm layer of Ag is electroplated on top of exposed gold and is chlorinated with FeCl_3_ solution to obtain a miniaturized 3.6 µm thick QRE (Figure 14b). Such a device is capable of offering stable and fully calibrated measurements of biomarkers in sweat, such as pH, Na^+^, and K^+^, in real time thanks to the presence of specific and selective polymeric membranes [88]. The pH sensitivity is 52 mV/pH with an ultra-low power consumption. Finally, the time stability of the QRE was tested after coating with polyvinyl butyral/NaCl matrix and by plotting the open-circuit potential while perfusing 23 mM NaCl through the channel as a function of time (versus a commercial flow-through Ag/AgCl reference electrode). As a result, a long-term (days to week) stable operation was demonstrated. Recently, Z. Xu et al. fabricated microscale (5 μm in lateral dimension) AgCl/Ag pseudo-reference electrodes together with silicon nanoribbon field-effect transistors (Si NRFETs) on the same sensor chip using fully CMOS-compatible process technology [89]. A 4 nm thick HfO_2_ passivation layer was deposited, serving as the pH sensing layer. Microscale pseudo-REs were obtained after the evaporation and chlorination of a 150 nm thick Ag layer on a 5 nm thick adhesion Ti layer located underneath it. Then, thorough electrical characterization of these p-REs was performed by measuring the open-circuit potential against a commercial Ag/AgCl RE in a 30 mM KCl solution at a pH of 7 in order to evaluate their long-term stability and reliability with and without the presence of a protective photoresistive layer. As a result, with the protective photoresistive layer, the long-term stability of the p-REs was recorded at over 40 h, with a low value for potential drift of 0.4 mV/h. In the absence of the resist protection, the AgCl/Ag p-RE lost electrical connection after about 30 min due to the peeling of the unprotected p-RE under fluid flow. The final sensors were used either in microfluidic or static configurations, demonstrating rapid stabilization and long-term stability [89]. Elsewhere, in view of enhancing the miniaturization, portability, manufacturability, and reliability of extended-gate FET (EGFET) pH sensors, which are crucial criteria in medical, agricultural, and environmental applications, another recent approach was explored by J.T. Yeh et al. [63]. The authors integrated Au electrodes (geometry not described) instead of planar Ag/AgCl reference electrodes. This strategy should be more cost-effective compared to the solid-state Ag/AgCl deposition process. The integrated Au electrode configuration enabled them to achieve a 96 mV/pH sensitivity with a 97% linearity across a pH range of 4 to 10. Stability tests revealed that the integrated Au electrodes had a low drift under different pH conditions for 16 min [63].

Summarizing this section on the Ag/AgCl pseudo-reference electrodes in Si NWFETs, numerous studies have focused on this type of electrode. They are easier to use than the external standard and are commercially available as Ag/AgCl reference electrodes. Regarding the two existing configurations, in comparison with the Ag/AgCl wire configuration, the solid-state integrated planar Ag/AgCl appears to be generating increasing interest. This is particularly confirmed in the field of purely electrochemical sensors, while this development is more recent for Si NWFETs. This trend is explained by the increasing development of portable and wearable biosensors. The Ag/AgCl layer fabrication involves Ag film deposition on the chip by several different techniques (evaporation, electroplating, printing, etc.), followed by its chlorination. However, sensing issues can occur when this electrode layer is no longer stable. Indeed, its robustness and long-term stability are the main problems, as the AgCl layer tends to dissolve gradually in the electrolyte, thus compromising the sensor’s reliability. As the electrode is integrated in the chip, it is not accessible enough to easily replace. This can be a problem when it comes to industrialization, since the entire chip would have to be changed due to the reference electrode failure. Consequently, several recommendations should be kept in mind to optimize these systems. First, given that the solid-state Ag/AgCl planar electrode is sensitive to variations in the Cl^−^ ion concentration in the electrolyte, it is important to keep this concentration constant and avoid environments with variable chloride concentrations. Second, where possible, the use of a coating membrane (PVB or Nafion) could help to preserve the electrode quality and slow its degradation. Furthermore—if still possible—to guarantee its proper operation and, therefore, its quality, it is important to monitor the open-circuit potential drift, which should be as low as possible. Thus, while the use of this integrated Ag/AgCl layer seems to be very attractive, it is subject to environmental controls. Alternatively, using an easily replaceable Ag/AgCl wire could also provide some advantages.

To conclude this part, the overview of the various methods of using Ag/AgCl reference electrodes in Si NWFET devices shows that two main strategies are chosen: either using an external conventional Ag/AgCl reference electrode (involving a saturated KCl salt solution) or using a Ag/AgCl pseudo-reference (without KCl solution). Table 2 illustrates these two strategies with their advantages, limitations, and applications.

In the following section, we address the importance of the reference electrode and the various biasing conditions of FET devices. In particular, we will review studies that have investigated the impact of these conditions on sensor sensitivity and signal drift.

## 3. Various Configurations of Device Biasing Using Ag/AgCl Reference Electrodes

While the previous section focused on the various configurations of the Ag/AgCl reference electrode in Si NWFETs, this section examines how some results can vary depending on these configurations. Usually, Si NWFETs are used in a back-gate configuration, where the silicon substrate is used as the gate for the device. However, it has been shown that working in a back-gate configuration while having a sensing solution on top of the nanowires can lead to very unreliable and unpredictable results [41]. The use of a reference electrode makes it possible to stabilize the potential in the liquid, thus avoiding or reducing the signal drift over time [15], and overcoming the lack of reliability and reproducibility of the signal. In addition, used wisely, REs make it possible to increase the sensitivity of the sensor by involving capacitive coupling effects within the device, as will be described further. This last configuration is referred to as dual-gate biasing. The effect of these different configurations on some device characteristics, such as the sensitivity and noise, are reported in the following examples. It can be seen that, depending on the studies, the effects are not similar. Indeed, many parameters are involved (FET device characteristics, electrolyte parameters, type of reference electrode, etc.), which are not always completely reported, making it difficult to conduct a rigorous comparison between the examples.

### 3.1. Effect on the Sensitivity

**Figure 15:**
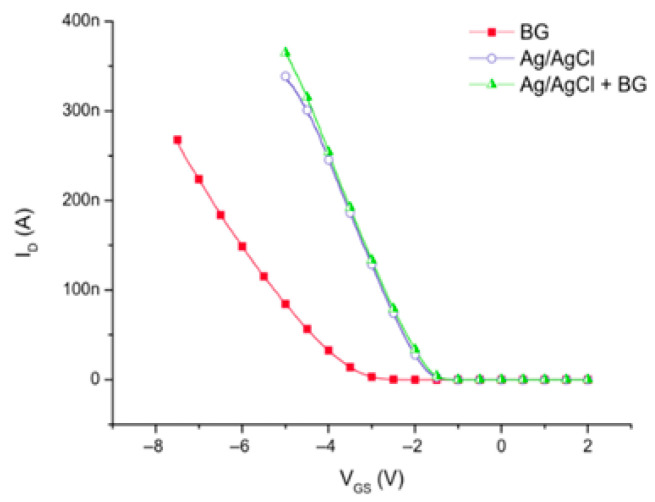
Comparison of I_D_-V_GS_ characteristics of a device when exposed to water for different modes of gate biasing: back-gate only (red squares), front-gate with back-gate voltage grounded (blue circles), and via back-gate and front-gate simultaneously (green triangles). Reprinted from Reference [90]. Available under CC BY 4.0 license.

Two comparatives studies have been carried out by M. Mescher et al. [90] and E. Accastelli [15] on the different gating possibilities. In those studies, the effect of back-gating, front-gating, and simultaneous gating via back- and front-gating are discussed. M. Mescher et al. [90] compared three types of biasing configuration on Si NWFETs (p-doped nanowires with a length of 3 μm, width of 300 nm, and height of 40 nm, covered with a silicon dioxide gate oxide with a thickness of 8 nm). The liquid solution was deionized water and a Ag/AgCl electrode (type not described) was placed at a fixed position in the solution. Firstly, the gate potential was applied only through the back-gate voltage. Secondly, the gate potential was applied through the reference electrode, with the back-gate being grounded. Lastly, the gate potential was applied through both the reference electrode and the back-gate (Figure 15).

These different transfer characteristic curves first show that every configuration allows the transistor to be switched on and work in depletion mode, as expected for p-type nanowires. Moreover, it can be noted that, when using the RE for gate biasing, the required potentials are lower than the ones used in back-gating, thus preserving the device in time and increasing its lifetime [90]. For that reason, front-gating could be more interesting, as also confirmed in another study [76]. Furthermore, in this study, using both the back-gate and the front-gate simultaneously showed no significant influence on the device’s characteristics. However, this result is different from the following ones.

**Figure 16:**
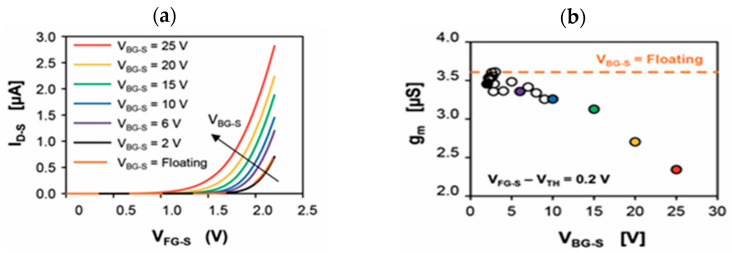
(**a**) Drain current (I_D-S_) versus front-gate voltage (V_FG-S_) for different back-gate voltages (V_BG-S_) [15]. Reprinted with the permission of the authors. (**b**) Transconductance (gm) versus back-gate voltage (V_BG-S_) for constant front-gate voltage potentials [15]. Reprinted with the permission of the authors.

E. Accastelli et al. [15,78] discussed the impact of simultaneous front-gating and back-gating on Si NRFETs. The Si nanoribbons (NRs) were fabricated from silicon-on-insulator (SOI) wafers, with the Si body covered by a high-quality 3 nm SiO_2_ gate oxide grown by thermal dry oxidation. The Ag/AgCl pseudo-reference electrodes used were obtained from anodization in 0.1 M KCl solution of silver tubes (Figure 9), as described in Section 2.3.1. The drain current I_D_ versus front-gating V_FG-S_ was studied for different back-gate voltages V_BG-S_ (Figure 16a). Meanwhile, the effect of the back-gate voltage on the transconductance (g_m_) was also studied (Figure 16b) to understand the impact of the back-gate voltage potential.

As can be seen from Figure 16a, working with high back-gate voltage potentials up to 25 V allows for the threshold voltage to be lower. However, working at really high potentials can be damaging to the device [15]. Moreover, the transconductance g_m_ (Figure 16b) significantly decreases with V_BG-S_ [15]. The sensitivity of the device being directly proportional to the transconductance, it is necessary to maximize it and thus to work with very low back-gate potentials. This could indicate a capacity to work at a floating back-gate potential so to maximize the transconductance. However, working with a floating back-gate can lead to many problems, such as increased low-frequency noise, which is to be avoided [91]. Therefore, the best configuration is to work with a front-gate biasing, while keeping the back-gate potential grounded, as shown by E. Accastelli et al. [78].

Thus, as can be observed in this last example, one can achieve an enhancement of the sensing sensitivity in the case of Si NWFETs on the SOI substrate by relevantly tuning the values of both V_BG_ and V_Ref_ (dual-gate) biasing configurations. As a result, the obtained sensitivity can reach values beyond the Nernst limit sensitivity of −59.2 mV/pH at 298K [21].

It is recalled that this Nernst limit originates from the hydroxylation of the sensing oxide surface interacting with the hydrogen ions in the solution. This creates a surface potential Ψ_o_ that is linked to the pH, according to the detailed analysis given by P. Bergvelt [21], leading to

$$\frac{d\Psi_{0}}{dpH}=-2.3\;\alpha \frac{K_{B}.T}{q}$$

where α is a dimensionless parameter, linked to the interface proton activity, varying between 0 and 1, *K_B_* is the Boltzmann constant, *q* is the elementary charge, and *T* is the absolute temperature.

If α = 1, at room temperature, the device reaches the so-called Nernstian sensitivity of 59.2 mV/pH, which is also the maximum achievable sensitivity. The dual-gate structure of Si NWFETs involving the BOx layer and the sensing oxide film (see Figure 1) enables us to overcome the Nernst sensitivity limit. Indeed, while fixing V_Ref_ and sweeping V_BG_ to obtain the pH-dependent shift in the transfer curves, a capacitive coupling between the usually thin oxide top gate film and the thick BOx gate can be involved according to a simplified relation [92,93]:

$$\frac{{\Delta V}_{BG}}{\Delta pH}=59.2\frac{C_{OX.DL}}{C_{BOx}}$$

where *C_OX.DL_* is the double-layer capacitance at the electrolyte/oxide interface in association with the thin oxide layer capacitance Cox; *C_DL_* is assumed to be much larger than *C_DL_* and can be neglected; *C_BOx_* is the BOx capacitance.

If the quality of the gate oxide and the BOx are similar, with almost the same dielectric constant, the term *Cox*/*C_BOx_* becomes an amplification factor providing enhanced sensitivity when *Cox* >> *C_BOx_*. Thus, the sensitivity is approximately amplified by the thickness ratio of thick BOx to thin gate oxide.

This geometry-dependent capacitive division has been well investigated by O. Knopmacher et al. [94] in Si NWFETs using a Pt reference electrode. H. J. Jang et al. [61] have also studied the effect of each parameter on the pH sensitivity using a commercial Ag/AgCl electrode with an ISFET. The ISFET characteristics are a 10 µm (length) × 10 µm (width) Si channel covered by a thin nm range thick SiO_2_ layer (front gate) on a SOI substrate with a 224 nm thick BOx. Transfer curves were investigated by sweeping V_BG_ at different fixed values of V_Ref_. An enhancement of sensitivity could be reached by decreasing the thickness of the Si channel from 85 nm to 4.3 nm; the sensitivity increased by more than twofold, reaching a value 426 mV/pH. Moreover, greater stability improvements could be obtained in this geometrical architecture. Elsewhere, due to this strong electrostatic coupling between the front-gate and back-gate, G. T. Ayele et al. [75] obtained ultra-high-sensitive and ultra-low-power CMOS-compatible pH sensors that were developed in the back-end-of-line (BEOL) of industrial 28 nm ultra-thin body and buried oxide (UTBB) fully depleted silicon-on-insulator (FDSOI) transistors. With aluminum oxide (Al_2_O_3_) deposited by atomic layer deposition (ALD) as a pH sensing film, the authors reported a sensitivity of 475 mV/pH and 730 mV/pH (12 times higher than the Nernst limit) in the extended gate and BEOL configuration, respectively.

R. Midahuen et al. [65] reported on Si NWFETs with a commercial miniaturized Ag/AgCl reference electrode, as described in Section 2.1.2, in a dual-gating configuration. Various Si nanochannels covered by a double oxide layer (4 nm thick HfO_2_ over a 3 nm thick SiO_2_) on SOI wafers were studied. They presented different Si architectures, such as NWs, nanoribbons (NRs), and honeycombs (HCs), with various sizes (lengths varying from 0.1 µm to 10 µm, widths varying from 30 nm to 1 µm, and thicknesses varying from 6 nm to 30 nm). It has been shown that, in the case of Si NR, by fixing the reference electrode at 0.25 V and by sweeping the back-gate voltage from 4 to 12 V, the pH sensitivity was enhanced by a factor 10, with a pH sensitivity up to 600 mV/pH.

Regarding the effect of the chemical nature of the sensing oxide thin film on the pH sensitivity, J. W. Kang et al. [62] compared the sensing properties of various high-dielectric-constant k-sensing oxide layers or membranes, such as SnO_2_, HfO_2_, ZrO_2_, and Ta_2_O_5_, as well as SiO_2_ and Si_3_N_4_. The devices were poly-Si-based dual-gate FETs fabricated as transducers to reduce the manufacturing costs compared with conventional silicon on insulator (SOI)-based dual-gate FETs. In addition, a separate detector (extended gate (EG)) was fabricated on a cheap glass substrate. Adapting the dual-gate structure and using a standard commercial Ag/AgCl reference electrode, the highest sensitivity of 478 mV/pH was obtained for the Ta_2_O_5_ film.

Other studies also break through the barrier of the Nernst response, showing outstanding sensitivities up to 2.25 V/pH [87]. The authors used a ZnO ISFET based on the dual-gate effect. A passivating self-assembled monolayer (SAM) of octadecylphosphonic acid was deposited on top of the semiconducting ZnO layer. The potential of the electrolyte V_ref_ was set by a KCl-buffered Ag/AgCl reference electrode. Particularly, the sensitivity scaled linearly with the ratio between the top and bottom gate capacitances.

Elsewhere, external parameters, such as the interface between the reference electrode and the electrolyte, the electrolyte itself, or the top oxide–electrolyte interface, are also involved, as is well modeled by J. Go et al. [95]. The authors studied the effects of such parameters on both the sensitivity and the signal-to-noise ratio. This last characteristic is also a crucial parameter that is not fully controlled. While its effects can be reduced and minimized in the case of pH measurements, they become non-negligible in the case of biomolecule detection, where the electrolyte Debye screening length often restricts the measurements, resulting in challenging studies with poor reproducibility.

**Figure 17:**
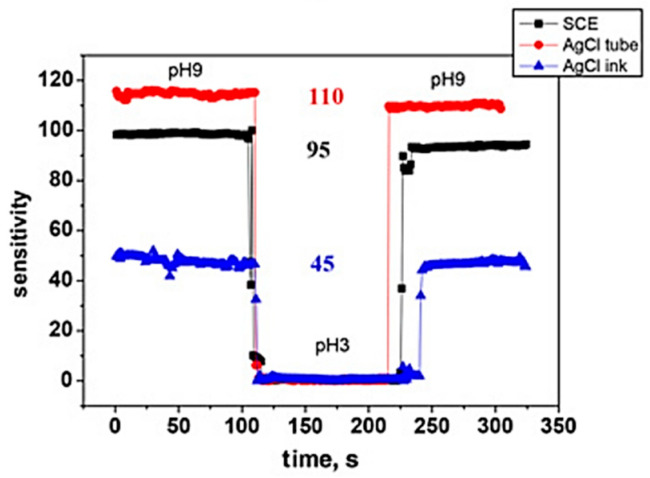
Normalized sensitivity value (ΔI/I0) according to the variation in pH concentration resulting from measurements of the real-time average value of Ids measured with two similar sensors and with three different reference electrodes: SCE-saturated calomel reference electrode, Ag/AgCl electrode (tube type), and integrated Ag/AgCl (ink) electrode. Reprinted from Ref. [64]. Available under CC BY 4.0 license.

Regarding the effect of the kind of reference electrode on the sensitivity, an interesting study was conducted by Y.T. Seo et al. [64], aiming to demonstrate how the choice of the reference electrode affects the pH measurement results obtained with a nanoFET device (Figure 6a). Three types of reference electrodes were used to establish this comparison: a saturated calomel reference electrode (SCE), a Ag/AgCl tube (tube-type), and an on-chip-integrated Ag/AgCl ink electrode, as illustrated in Section 2.1.2 (Figure 6b). In the last configuration, the integrated reference electrode was fabricated by applying and drying a Ag/AgCl paste on the FET device. For each reference electrode, an evaluation of the pH sensitivity (at a pH of 9 and pH of 3) as a function of time was performed and a comparison between the equivalent surface potentials was made. The experimental results (Figure 17) show that the three electrodes lead to different values of sensitivity for the same pH value.

As can be observed, careful selection of the reference electrode is needed to accurately control the reference voltage of the solution when measuring the potentials of biological materials. The RE has a significant impact on the sensing measurement when the ion concentration is diluted. Ag/AgCl tubing seems to be the best option but is not so different from SCE. However, it has been mentioned earlier by J. Zhou et al. [50] that Ag/AgCl electrodes are the most convenient for Si NWFETs. Technical solutions have been proposed to replace the standard and bulky Ag/AgCl reference electrode, which hinders the commercialization of ISFETs and NWFETs due to its dimensions and incompatibility with CMOS technology.

### 3.2. Effect on Noise

The resolution and sensitivity of BioFET sensors can be affected by both biochemical noises and internal electrical noises. The chemical noise is associated with the random binding/unbinding of H^+^ ions on the sensing layer, while the FET underlying any ISFET is the source of the electrical noise. Obviously, these effects depend on the device architecture [96]. The downscaling of Si NWFETs while maintaining the quality of the device surfaces leads to an increase in low-frequency noise due to a decreasing number of charge carriers and increasing surface contributions to electric noise [97].

**Figure 18:**
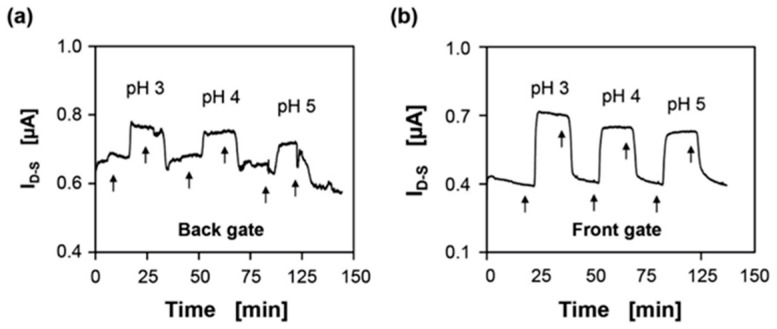
Drain current measurement in back-gate (**a**) and liquid-gate (**b**) configurations. The arrows indicate the injection of the solution [15]. Reprinted with the permission of the authors.

E. Accastelli et al. [78] have shown that working in a front-gate configuration leads to a reduced noise in pH measurements in real time compared with a back-gate configuration (Figure 18a,b). Through the back-gate polarizing of Si NR without any front-gate contact (electrolyte solution left electrically floating), the device current is subjected to noise, particularly when changing the buffer solution by injection in the microfluidic channels (Figure 18a). On the contrary, the front-gating configuration with the grounded back-gate leads to an improved robustness against instabilities and noise by preventing parasitic coupling between the electrolyte solution and the environment (Figure 18b). Here, pseudo-Ag/AgCl reference electrodes were used and fabricated from silver tubes with a 5 µm thick AgCl metal salt layer [78], as previously described in Section 2.3.1.

F. Bellando et al. [89] performed a careful and systematic analysis of pH sensitivity and noise measurements of ISFETs with a home-made integrated miniaturized quasi-reference electrode (QRE), as described in Section 2.3.2. They were able to emphasize different drain current regimes, where the contributions of the different noises are different. However, an additional and significant current noise found at the reference electrode could not be excluded.

**Figure 19:**
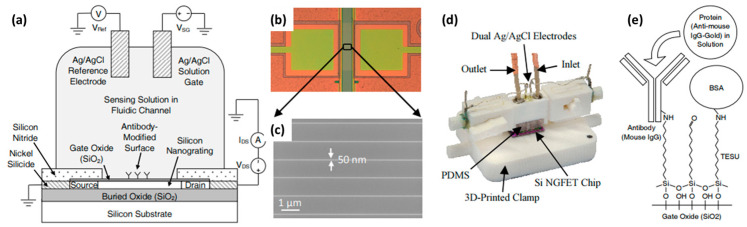
(**a**) Schematic of protein sensing with a Si-nanograting FET (NGFET) and dual Ag/AgCl electrodes. The oxide–electrolyte surface of the nanograting is functionalized with antibody. The FET is biased in subthreshold region by a solution gate at the top of the fluidic channel. The other electrode monitors the bulk solution potential. (**b**) Optical image and (**c**) electron micrograph of a typical Si NGFET with 100 nanowires in the grating. Each nanowire is 50 nm in width, 30 nm in height, and 20 µm in length. (**d**) Three-dimensionally printed clamp for protein sensing with dual Ag/AgCl electrodes. (**e**) Species at the gate oxide surface after modification with TESU, antibody (mouse IgG), and passivation (with BSA). The corresponding antigen used in this study is anti-mouse IgG with gold. Reprinted with permission from Reference [98]. Copyright © 2014 IEEE.

An alternative and widely accepted solution is to use two FETs. One is the bioFET, while another one is a reference FET (REFET), which is non-responsive to the studied analyte. This system enables differential measurement between the bioFET and the REFET, eliminating the effects of unstable electrode/electrolyte potential, which appears as a common signal in the output of both FETs. The noise resulting from the electrolyte potential, which is amplified together with the small analyte signals, becomes an issue in certain biosensing applications, particularly when the analyte concentration is extremely low (order of fM) and varies over time [98]. A well-known example is the direct detection of penicillin with enzymatically modified ion-sensitive FET (ENFET) by S. Caras et al. [99]. In this case, the ENFET probe consisted of two separate ISFETs, with one gate having a cross-linked albumin–penicillinase membrane (penicillin-sensitive gate) and the other gate having only a cross-linked albumin membrane and exhibiting only a pH response (reference gate). Thus, the differential mode of operation provides an automatic compensation of the changes in ambient pH and temperature. This is important for operation in a low buffer capacity, where the sensitivity is the highest [99]. It is to be noted that the use of REFETs remains very challenging because it requires a careful surface modification. Indeed, it needs to be both chemically inert to the analyte while at the same time as sensitive as the bioFET to other electrolyte changes (e.g., pH, temperature, ionic strength, etc.) [98]. To overcome this REFET challenge, instead of using a REFET, P. Zang et al. [98] had the idea to use a second Ag/AgCl reference electrode to monitor and suppress the electrolyte potential noise. This kind of experiment was reported for the first time in 2014 in the case of dynamic measurements to study the time-dependent protein sensing tests. A diagram of the setup is presented in Figure 19. It is to be noted that the kind of Ag/AgCl reference electrode is not described in the paper. The authors performed the detection of antibody–antigen (anti-mouse IgG) binding detection using Si NWFETs. Two identical Ag/AgCl electrodes were mounted on top of a fluidic channel. Their role is different: the first one is the solution gate that sets the electrolyte potential and the other one is used as a reference electrode to monitor the electrolyte potential. Such a configuration allows the noise in the unstable electrolyte potential to be reduced and provides an increased readability with a reduced signal level variation. With this method, the limit of detection is reduced by 50–70% [98].

Especially during dynamic measurements, a streaming potential is generated by the fluid flow of the electrolyte solution on the sensitive surface in the microfluidic channel. In comparison with static measurements, the distribution of ions and counterions in the electrical double layer (EDL) is not equivalent between two points along the flow direction, resulting in a potential difference that is also called the streaming potential. Moreover, fluctuations in the fluidic flow rate during sample switching reduce the quality and reliability of the measurement system. All of these contributions induce noise and erroneous signal generation [100]. Along with that, it was also concluded that the streaming potential could change the conductance of FET-based biosensors, such as SiNW-bioFET. At the same time, it was concluded that the configuration of the reference electrode strongly influences the effect of the streaming potential [100]. Thus, J. Lee et al. [101] thoroughly investigated the problem of the streaming potential (V_str_) generated in the microfluidic channel. They succeeded in reducing the effect of the streaming potential thanks to two experimental characteristics: the use of two Ag/AgCl electrodes and a microfluidic channel coated with a thin Ag layer. The Ag/AgCl wires were inserted into the tubes as pseudo-reference electrodes in order to control the electrical potential of the electrolyte solution located at the inlet and outlet of the microfluidic channel, respectively. The metal layer in the PDMS microfluidic channel set a constant electrical potential along the path of the fluid for a given reference electrode voltage, regardless of the flow velocity, resulting in a reduction in noise by four orders of magnitude.

Summarizing this biasing section, the studies show that, beyond the correct use of the Ag/AgCl reference electrode (including its own characteristics, its potential value, its positioning, etc.), optimizing the device architecture is crucial in order to enhance the sensitivity. Toward this aim, some technological strategies are emphasized. First, an excellent strategy is to operate in dual-gate biasing, which consists of the simultaneous application of voltages to the back- and liquid-gates. This enables us to benefit from an effective electric gain involving the capacitance ratio of both the top thin oxide and the thick BOx. Therefore, the thermodynamic Nernst limit can be overcome. In the case of the impossibility of dual-gate biasing, it is preferable to operate with front-gate biasing while keeping the back-gate grounded for low noise and high transconductance. The floating back-gate should be avoided due to 1/f noise (a type of low-frequency noise that increases in strength as the frequency decreases and which limits the performance). Second, the choice of the top thin oxide nature is important, emphasizing that high-dielectric-constant oxides such as HfO_2_ and Ta_2_O_5_ are preferable for pH measurements due to their high capacity for hydroxylation.

Finally, it is also to be noted that, even though a front-gate configuration is highly recommended, there are still numerous studies where no reference electrode is used in Si NWFET biosensors, as reported by B. M. Lowe et al. [11].

## 4. Conclusions

A stable reference electrode is essential for ISFETs and Si NWFET-based sensors in order to provide a stable and reliable solution potential during measurements. This improves the accuracy and sensitivity of the sensor while reducing the noise and potential drift. In this review, starting from the systematic use of the Ag/AgCl reference electrode in the three-electrode cells of conventional electrochemical sensors, we presented the various methods of using and integrating this type of reference electrode in Si NWFET sensors with their associated challenges.

Two main usage strategies for Ag/AgCl reference electrodes in Si NWFETs for biosensing have emerged. They include the use of either a standard, external, and commercially available Ag/AgCl reference electrodes or a pseudo-reference Ag/AgCl electrode. Their specificities are summarized in Table 2, together with their advantages, drawbacks, and application fields. If the standard Ag/AgCl reference electrode is still required for precise calibration in laboratory tests, the trend goes towards the use of pseudo-reference Ag/AgCl electrodes, and notably integrated solid-state Ag/AgCl electrodes. Their advantages are as follows: (i) to avoid the bulky presence of the standard Ag/AgCl reference electrode with the presence of saturated KCl solution, and therefore the leakage risk; (ii) process compatibility between their fabrication step and chip manufacturing. Their fabrication, which has been under study for a long time in pure electrochemical sensors, has been studied more recently but less explored in Si NWFETs. Nevertheless, they present several drawbacks, compromising the sensitivity and time stability of the sensor, i.e., a short lifespan due to the gradual dissolution of the AgCl layer and sensitivity to electrolyte Cl^−^ ions. Some solutions have been proposed, such as environment control and the use of a protecting membrane. Moreover, given that the electrodes are directly on the chip, they cannot be easily replaced. Alternatively, the use of an easy-to-handle Ag/AgCl wire pseudo-reference offers an advantage. In the event of degradation, it is easier to replace the wire with a new one, which is not possible with planar solid-state electrodes.

Some specific biasing conditions have emerged, enabling the enhancement of the Si NWFET sensitivity. Particularly, a powerful technological strategy is the dual-gate mode, involving both the reference electrode voltage and the back-gate voltage. This mode relies on an optimized Si NWFET architecture that involves capacitive coupling between the thin top oxide layer and the thick BOx. In addition, the nature of the top oxide is of importance.

By classifying the various methods of using Ag/AgCl reference electrodes in Si NWFETs, this review has emphasized that the whole system—Si NWFET/electrolyte/reference electrode—should be carefully described, including the characteristics of each of these three parts, as is suggested in Table 1. This is an important aspect for future rigorous comparative studies of Si NWFET sensing performances.

Because the field of portable and wearable sensors is rapidly expanding, the need for completely integrated and robust reference electrodes in Si NWFETs is important. Therefore, it should be of great interest to continue developing and optimizing their integration in Si NWFETs. This would enable us to improve the signal in term of sensitivity, reliability, and reproducibility. The challenge would consist in improving and controlling the characteristics of the solid-state Ag/AgCl pseudo-reference electrodes in terms of lifespan, time stability, environmental control, and protective membrane investigation. Therefore, their fabrication process should be deeply investigated and characterized. The investigations should focus on the Ag deposition, chlorination process, SU-8 passivation with eventual membrane protection, as well as the dissolution failure process. Useful help could probably be found using machine learning, as in the study of N. Ayadi et al. [102] for predictive models concerning the effects of Ag/AgCl reference electrode characteristics on the signal. This should include the impact of the reference electrode on the analyte and any contamination that may be introduced into the system.

## Figures and Tables

**Table 1:** Proposal of all important parameters to be taken into account and mentioned in any publication on Si NWFET transistors for biosensing.

Si NWFET Characteristics	Electrolyte	Reference Electrode	Biasing Conditions
Si NW geometry: length, thickness, width Si NW doping Top oxide layer: nature, thickness Functionalization layer (if it exists): nature	Nature Concentration	Type Position in the device	V_BG_ V_Ref_ V_s_ and V_D_

**Table 2:** Summary of advantages, drawbacks, and application fields of the various kinds of Ag/AgCl reference electrodes in Si NWFETs.

Ag/AgCl Electrode Type		Advantages	Drawbacks	Applications
**Reference electrode: with KCL salts**	Standard external electrode with liquid KCl [53,54,55,56,57,58,59,60,61,62,63,64,65,66,67]	High stability Easy to calibrate	Bulky, no integration High cost	Laboratory tests Static configuration
	Integrated electrode with liquid/solid KCl [68,69,70,76]	Good stability Miniaturization	Complexity of fabrication Leak risk	Portable devices Microfluidic
**Pseudo- or quasi-reference electrode: without KCL salts**	External wire [15,51,68,77,78,79,80]	Low cost No leak risk Easy manipulation Partial integration Easily replaceable	No complete integration Electrolyte Cl^−^ concentration dependence	Portable devices Microfluidic Disposable sensors Controlled environment
	Integrated planar [81,82,83,84,85,86,87,88,89,90]	Low cost No leak risk Complete integration during fabrication process	Electrolyte Cl^−^ concentration dependence Progressive dissolution of AgCl layer Potential drift risk Not replaceable	

## Data Availability

No new data were created.
